# Enrichment of hepatic glycogen and plasma glucose from H₂^18^O informs gluconeogenic and indirect pathway fluxes in naturally feeding mice

**DOI:** 10.1002/nbm.4837

**Published:** 2022-10-22

**Authors:** Margarida Coelho, Rohit Mahar, Getachew D. Belew, Alejandra Torres, Cristina Barosa, Fernando Cabral, Ivan Viegas, Amalia Gastaldelli, Vera M. Mendes, Bruno Manadas, John G. Jones, Matthew E. Merritt

**Affiliations:** ^1^ CNC ‐ Center for Neurosciences and Cell Biology University of Coimbra Coimbra Portugal; ^2^ Department of Chemistry, Faculty of Sciences and Technology University of Coimbra Coimbra Portugal; ^3^ Department of Biochemistry and Molecular Biology University of Florida Gainesville Florida USA; ^4^ Center for Functional Ecology, Department of Life Sciences University of Coimbra Coimbra Portugal; ^5^ Institute of Clinical Physiology, CNR Pisa Italy

**Keywords:** fructose, gluconeogenesis, glycogenesis, isotope shift, triose phosphates

## Abstract

Deuterated water (^2^H_2_O) is a widely used tracer of carbohydrate biosynthesis in both preclinical and clinical settings, but the significant kinetic isotope effects (KIE) of ^2^H can distort metabolic information and mediate toxicity. ^18^O‐water (H_2_^18^O) has no significant KIE and is incorporated into specific carbohydrate oxygens via well‐defined mechanisms, but to date it has not been evaluated in any animal model. Mice were given H_2_^18^O during overnight feeding and ^18^O‐enrichments of liver glycogen, triglyceride glycerol (TG), and blood glucose were quantified by ^13^C NMR and mass spectrometry (MS). Enrichment of oxygens 5 and 6 relative to body water informed indirect pathway contributions from the Krebs cycle and triose phosphate sources. Compared with mice fed normal chow (NC), mice whose NC was supplemented with a fructose/glucose mix (i.e., a high sugar [HS] diet) had significantly higher indirect pathway contributions from triose phosphate sources, consistent with fructose glycogenesis. Blood glucose and liver TG ^18^O‐enrichments were quantified by MS. Blood glucose ^18^O‐enrichment was significantly higher for HS versus NC mice and was consistent with gluconeogenic fructose metabolism. TG ^18^O‐enrichment was extensive for both NC and HS mice, indicating a high turnover of liver triglyceride, independent of diet. Thus H_2_^18^O informs hepatic carbohydrate biosynthesis in similar detail to ^2^H_2_O but without KIE‐associated risks.

## Graphical abstract

^18^O‐enrichment of liver glycogen, triglyceride glycerol, and blood glucose of mice given ^18^O‐water during overnight feeding was quantified by ^13^C NMR and MS. Glycogen ^18^O‐enrichment of oxygens 3, 5, and 6 from isotope‐shifted ^13^C signals informed indirect pathway contributions from different sources. Glucose ^18^O‐enrichment by LC–MS/MS yielded an all‐oxygens average, while GC–MS provided average estimates of oxygens 2–5, 2–4, and 4–6, evaluating gluconeogenesis. Triglyceride glycerol ^18^O‐enrichment, measured by GC–MS, indicated high liver turnover rates.

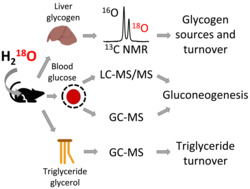

**Abbreviations used**
^2^H_2_O: water enriched with deuterium
DBS: dried blood spot
GC–MS: gas chromatography mass spectrometry
H_2_^18^O: water enriched with oxygen‐18
HS: chow supplemented with fructose/glucose mix (i.e., a high sugar diet)
KIE: kinetic isotope effects
LC–MS/MS: liquid chromatography coupled to tandem mass spectrometry
MAG: monoacetone glucose
NC: normal chow
TAMAG: 3,5,6‐tri‐O‐[1‐^13^C]acetyl monoacetone glucose

## 1 INTRODUCTION

Water enriched with ^3^H or ^2^H has been widely used for the study of biosynthetic activities in whole organisms, including the endogenous synthesis of glucose and glycogen.^1^, ^2^, ^3^, ^4^, ^5^ An important advantage of this approach over methods involving ^13^C‐labeled substrates is that labeled water rapidly distributes throughout the entire organism, thereby providing constant precursor enrichment for all tissues that can be easily measured from blood, saliva, or urine. Moreover, for both glucose and glycogen, the incorporation of water hydrogen into certain product positions is independent of precursor sources, while the enrichment of other positions is specific to a particular precursor source.^4^, ^6^, ^7^ Thus, this approach provides precise estimates of fractional synthetic or turnover rates of glucose or glycogen irrespective of the available precursors, while at the same time resolving the contributions of different precursors to the overall synthesis rate. The principal uncertainties associated with the use of hydrogen isotopes in this manner include significant discrimination against enrichment of certain product positions because of strong kinetic isotope effects (KIE).^8^ Discrimination of ^2^H incorporation from water into position 2 of glycogen has been observed in both mice and fish.^9^, ^10^ Nevertheless, methods for quantifying positional ^2^H‐enrichments of glucose and glycogen have been developed for both mass spectrometry (MS)^11^, ^12^ and nuclear magnetic resonance (NMR) spectroscopy.^7^, ^13^, ^14^, ^15^

**FIGURE 1:**
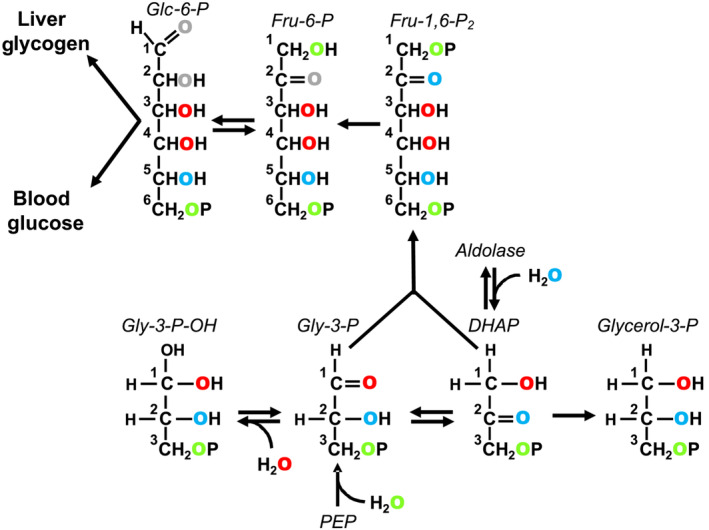
Incorporation of ^18^O from water into carbohydrate precursor metabolites at the level of phosphoenolpyruvate (PEP) and triose phosphates. Exchange of dihydroxyacetone phosphate (DHAP) with aldolase results in the incorporation of ^18^O into position 2 (shown in blue). DHAP derived from fructose via the aldolase‐mediated cleavage of fructose‐1‐phosphate will also have ^18^O incorporated into this position (not shown). Exchange of DHAP with glyceraldehyde‐3‐phosphate (Gly‐3‐P) mediated by triose phosphate isomerase and conversion of the triose phosphates to fructose‐1,6‐bisphosphate (Fru‐1,6‐P_2_) results in ^18^O incorporation into positions 2 and 5. The reversible hydration of Gly‐3‐P to form an acetal (Gly‐3‐P‐OH) results in the incorporation of ^18^O into position 1 of Gly‐3‐P (shown in red). Exchange of Gly‐3‐P and DHAP via triose phosphate isomerase and subsequent conversion to Fru‐1,6‐P_2_ via aldolase results in ^18^O incorporation into positions 4 and 3. Addition of water to PEP via enolase incorporates ^18^O into position 3 of Gly‐3‐P (shown in green). Triose phosphate isomerase exchange and aldolase generate Fru‐1,6‐P_2_ enriched in positions 1 and 6. The position 2 oxygen of fructose‐6‐phosphate (Fru‐6‐P) can undergo additional exchange with water via anomerization (shown in gray). Likewise, the position 1 oxygen of both glucose‐6‐phosphate (Glc‐6‐P) and glucose can be exchanged with water via anomerization (also shown in gray)

The oxygens of water also undergo exchanges and/or additions with precursors of glucose and glycogen synthesis, hence in the presence of ^18^O‐enriched water (H_2_^18^O), the hexose oxygens become enriched with ^18^O (Figure 1). In contrast to hydrogen isotopes, the much smaller relative mass difference between ^18^O and ^16^O means the absence of significant isotope discrimination because of KIE. We recently demonstrated ^18^O‐incorporation from H_2_^18^O into the oxygens of glucose‐6‐phosphate in hemolysate preparations that was in accordance with known exchange mechanisms acting at the triose and hexose phosphate levels.^16^ These measurements were performed by ^13^C NMR analysis of the monoacetone glucose derivative and quantification of the isotope‐shifted ^18^O‐signals. The objective of the present study was to translate this approach into animal models and test the applicability of H_2_^18^O as a tracer for hepatic carbohydrate biosynthesis. Because the ^18^O‐enrichment levels achievable in vivo are typically less than in vitro, we sought to increase the sensitivity of our ^13^C NMR measurements through a novel glucose derivative: 3,5,6‐tri‐O‐[1‐^13^C]acetyl monoacetone glucose (TAMAG). For this derivative, the number of ^13^C reporter nuclei for oxygens 3, 5, and 6 is amplified by a factor of nearly 100 over the background ^13^C level.

## 2 METHODS

### 2.1 Materials

D‐glucose was obtained from Sigma‐Aldrich (St. Louis, MO, USA). [2‐^18^O]glucose (> 90% enrichment), [5‐^18^O]glucose (> 90% enrichment), and [6‐^18^O]glucose (> 90% enrichment) were obtained from Omicron Biochemicals, Inc. (South Bend, IN, USA). [U‐^13^C_6_, U‐^2^H_7_]glucose (99% and 97%–98% enriched, respectively) was obtained from Cambridge Isotope Laboratories Inc. (Andover, MA, USA) and purchased through Tracertec (Madrid, Spain). ^18^O‐water at 97% enrichment was obtained from Sercon Ltd (Crewe, UK). Acetonitrile (LC–MS grade) and water (LC–MS grade) were acquired from VWR (Radnor, PA, USA), while ethanol (absolute [UV‐IR‐HPLC PAI]) was acquired from Fisher Chemical (Waltham, MA, USA). Hydroxylamine hydrochloride was obtained from Acros Organics from Thermo Fisher Scientific (NJ, USA). Propionic anhydride and pyridine were procured from Sigma Aldrich and Thermo Fisher Scientific, respectively. Silylation reagent (MTBSTFA + 1% TBDMCS) was purchased from Thermo Fisher Scientific (Waltham, MA, USA).

### 2.2 Animal studies

Animal studies were approved by the University of Coimbra Ethics Committee on Animal Studies and the Portuguese National Authority for Animal Health (approval code 0421/000/000/2013). All animal procedures were accomplished in full accordance with Portuguese National Authority for Animal Health guidelines and European regulations (European Union Directive 2010/63/EU). Adult male C57BL/6 mice were obtained from Charles River Labs (Barcelona, Spain) and housed at the University of Coimbra Biotech Bioterium, where they were maintained in a controlled environment, with a 12‐h light/12‐h dark cycle. Following delivery to the Bioterium, the mice were acclimatized for 2 weeks, with free access to water and standard chow. After the acclimatization period, eight mice were fed a normal chow (NC) diet and eight mice were fed a NC diet supplemented with drinking water containing a 55/45 fructose/glucose mixture at a concentration of 30% w/v (i.e., a high sugar [HS] diet) for 3 days. On the evening of the final day, a bolus of 97%‐enriched H_2_^18^O (8/100 g body weight) containing 9 mg/ml NaCl was administered with two intraperitoneal (i.p.) injections 2 h apart and the drinking water was enriched to 10% with H_2_^18^O. Mice were euthanized the next morning and biological fluids and tissues were collected. Arterial blood was collected and immediately centrifuged at 2000 × g for 5 min at 4°C and plasma was isolated and stored at −80°C for further experimental analysis, including for the analysis of body water ^18^O‐enrichment. Liver and other tissues, including adipose tissue depots, were freeze‐clamped and stored at −80°C until further processing.

### 2.3 Liver glycogen and triglyceride purification

Frozen liver powder from each animal (approximately 500 mg) was treated with methanol (4.6 ml/g) and methyl tert‐butyl ether (15.4 ml/g) for lipid extraction. Glycogen was extracted from the pellet by treatment with 30% potassium hydroxide (10 ml/g) at 70°C for 1 h. The mixture was treated with 8 ml/g 6% Na_2_SO_4_ and glycogen was precipitated with 50 ml/g ethanol. After centrifugation, the solid residue was resuspended in water and the pH was adjusted to 8. After drying, the pellet was resuspended in 5 ml of 0.05 M acetate buffer, pH 4.5. For glucose quantification, 100 μl of each sample was collected prior to glycogen digestion. To the remaining glycogen, 200 U of amyloglucosidase from *Aspergillus niger* (59.9 U/mg; Fluka, NC, USA) dissolved in the acetate buffer was added and incubated for 5 h at 55°C. After collection of 100 μl for glycogen quantification, the supernatant was deproteinized and evaporated. Glycogen was quantified by analyzing the glucose in both 100‐μl aliquots using a Miura 200 (ISE, S.r.l, Rome, Italy). Triglycerides were purified from the evaporated organic fraction with solid‐phase extraction as previously described.^17^ The triglycerides were then transesterified with sodium methoxide in methanol^18^ and the aqueous fraction containing glycerol was evaporated.

### 2.4 Blood sampling and processing

Blood was harvested without anticoagulants. A few microliters of blood was placed on 903 Protein Saver Snap Apart Card filter paper (Whatman, GE Healthcare Ltd, Cardiff, UK), which was then dried at room temperature and stored in a desiccator^19^ with the dried blood spots (DBSs) subjected to gas chromatography MS (GC–MS) and liquid chromatography coupled to tandem MS (LC–MS/MS) analyses. The remaining blood was centrifuged at 2862 × *g* for 5 min at 4°C to eliminate the cell debris. The sample was then deproteinized by the addition of 1.5 ml of ice‐cold 0.3 M ZnSO_4_ followed by 1.5 ml of ice‐cold 0.15 M Ba (OH)_2_ per ml of whole blood, with the resulting insoluble material precipitated by centrifugation. The supernatant was desalted by passage through a mixed‐bed ion exchange resin (5 ml of Amberlite and 2.5 ml of Dowex‐WX 8–200) followed by 15 ml of distilled water. The eluate pH was adjusted to 7 and lyophilized.

### 2.5 Determination of ^18^O body water enrichment

Plasma and urine samples were diluted 100‐fold to a final volume of 1 ml with water (LC–MS grade from VWR, Radnor, PA, USA). The enrichment of ^18^O‐water within these samples was determined with a model LWIA‐24d liquid water isotope analyzer (Los Gatos Research Inc., Mountain View, CA, USA).

### 2.6 Derivatization of glucose to TAMAG

Glucose from both liver glycogen and blood extracts was derivatized to TAMAG. Each lyophilized extract was vigorously mixed with 5 ml of acetone containing 4% sulfuric acid (v/v) for 8 h. The mixture was stirred overnight at room temperature to yield diacetone glucose. The acetonation reaction was quenched by adding 5 ml of water and the pH was adjusted to 2 with 1 M Na_2_CO_3_. The newly formed diacetone glucose was hydrolyzed to monoacetone glucose (MAG) by incubation at 40°C for 5 h. The solution pH was then increased to 8 with 1 M Na_2_CO_3_ and the samples were dried by rotary evaporation under vacuum. The MAG fraction in the residue was extracted with 4 ml of boiling ethyl acetate. Following evaporation of ethyl acetate, MAG was further purified by solid‐phase extraction using 500 mg of Discovery DSC‐18 column, eluted with 10% acetonitrile. For conversion of MAG to TAMAG, up to 2 mg of MAG was resuspended in 180 μl of dichloromethane and 9 μl of triethylamine. A 10.2‐μl portion of [1,1`‐^13^C_2_]acetic anhydride (Cambridge Isotope Laboratories, Inc., MA, USA) was added with continuous stirring at 25°C overnight. Removal of solvent and purification was performed by silica gel column chromatography with ethyl acetate‐hexane (1:7) elution. The evaporated residue was dissolved in deuterated acetone for ^13^C NMR analysis.

### 2.7 ^13^C NMR spectroscopy and data analysis

Proton‐decoupled ^13^C NMR spectra of TAMAG prepared from liver glycogen and blood glucose were acquired at 25°C with a Bruker Avance III 800 MHz NMR spectrometer (Billerica, MA, USA) operating at 201.16 MHz for ^13^C. A 5‐mm TXI cryoprobe was utilized to record ^13^C NMR data. ^13^C NMR spectra were obtained with a 45‐degree pulse, an acquisition time of 2.5 s, and an interpulse delay of 0.5 s. The number of acquisitions ranged from 2000 to 3000. All spectra were analyzed with ACD/NMR Processor 12 spectral analysis software (Advanced Chemistry Development, Inc., Toronto, ON, Canada). The relative areas of ^18^O‐isotopic‐shifted and parent ^13^C signals were calculated by peak fitting. Positional ^18^O‐enrichment (%) was calculated as follows:

PositionalO18enrichment%=Osignal area18Osignal area+Osignal area1816×100.

Excess ^18^O‐enrichment for each position was estimated by subtracting the background abundance (0.204%) from the measured positional enrichment.

The indirect pathway contributions to glycogen synthesis resolved into precursors entering at the levels of the Krebs cycle and triose phosphate pools were estimated from the ^18^O‐enrichments of glycogen oxygens 5, 6, and body water as follows:

Total indirect pathway%=Glycogen oxygen5O18enrichmentO18plasma water enrichment×100

Indirect pathwayKrebs cycle%=Glycogen oxygen6O18enrichmentO18plasma water enrichment×100

Indirect pathwayTrioseP%=Glycogen oxygen5─oxygen6O18enrichmentO18plasma water enrichment×100.

### 2.8 LC–MS/MS of DBSs and data analysis

To each 6‐mm DBS punch in a 1.5‐ml Eppendorf centrifuge tube, 50 μl of 2% acetonitrile containing 30 μM [U‐^13^C_6_, U‐^2^H_7_]glucose as an internal standard was added. To this, 450 μl of ethanol was added and the tube was placed in an ultrasonic bath for 45 min. Following a 5‐min centrifugation at 14,100 × *g*, the supernatant was evaporated. Samples were reconstituted in 50 μl of H_2_O and sonicated using a cup‐horn sonicator for 2 min (40% amplitude with 1‐s pulses and 1‐s pauses). The samples were further cleaned by solid‐phase extraction using 100‐μl C18 pipette tips from Agilent Technologies (Santa Clara, CA, USA). The tips were wetted with 40 μl of 50% acetonitrile and equilibrated with 120 μl of 2% acetonitrile. Fifty microliters of each sample was applied to the C18 tip and the flow‐through was collected. The tip was then washed with 50 μl of 2% acetonitrile, which was collected in the same tube as the sample. The final volume consisted of 100 μl for an injection volume of 1 μl. Standard solutions and mixtures of glucose analytes were prepared in 2% acetonitrile, and calibration curves were constructed for unlabeled glucose and [2‐^18^O]‐, [5‐^18^O]‐, and [6‐^18^O]glucose with working ranges spanning 15–240 μM for unlabeled glucose and 0.4–15 μM for the ^18^O‐enriched glucoses. Each standard was spiked with the same amount of internal standard (30 μM [U‐^13^C_6_,U‐^2^H_7_]glucose), final concentration 15 pmol/μl.

Samples were analyzed on an LC Nexera system (Shimadzu, Kyoto, Japan) coupled to a hybrid triple quadrupole/linear ion‐trap 4000 QTrap mass spectrometer operated by Analyst 1.6.3 (AB Sciex, Framingham, MA, USA). The injector was a CTC‐xt (PAL System, CTC Analytics AG, Zwingen, Switzerland). Chromatographic separation was performed with a Luna Amino 100 Å, 3‐μm, 150 × 2.0 mm column (Phenomenex, Torrance, CA, USA) with a 4 × 2.0 mm NH_2_ guard‐column (Phenomenex). The flow rate was set to 150 μl/min and the mobile phases A and B were water and acetonitrile, respectively.

The LC was programmed as follows: 80%–30% of B (0–9 min), 30%–20% of B (9–10 min), 20% of B (10–11 min), and 20%–5% of B (11–12 min). The ionization source ESI Turbo V was operated in the negative mode set to an ion spray voltage of 4500 V, 35 psi for nebulizer gas 1, 0 psi for the nebulizer gas 2, 30 psi for the curtain gas, and a temperature of 450°C. All molecules were analyzed by multiple reaction monitoring (MRM) settings Q1 and Q3 at unit resolution, the entrance potential at −4 eV, and the collision gas was at −3 psi. The MRM detection window was set to 1235 ms and the target scan time to 60 ms. The collision energy was set at −14 eV and declustering potential at −50 eV for all transitions. This method has been previously validated for analysis of [U‐^13^C]‐ and [6,6‐^2^H]glucose enrichments in DBSs of rats administered with these tracers.^19^

Peak area ratios were calculated from integrated peak areas of the analyte and internal standard on MultiQuant 2.1.1 (AB Sciex). Quantification was performed for glucose M + 0, M + 2, M + 4, and M + 6, by monitoring the sum of the transitions of each glucose species (Table S1). Enrichment of glucose M + 2 was corrected for background isotope contributions using data from the glucose M calibration curve.

Fractional ^18^O enrichment (FE) was calculated for each glucose species as follows:

FE%=Opeak area ratio ofM+x18∑Opeak area ratio ofallglucose species18×100.

Average fractional enrichment was calculated for each sample as follows:

AverageO18FE%=[M+2OFE]+[M+4OFE×2]+1818M+6O18FE×3Total number of carbons×100,

where the M + 4 and M + 6 signals are assumed to represent glucose molecules containing two and three ^18^O, respectively.

The fraction of plasma glucose derived from gluconeogenesis was calculated for each sample as follows:

Gluconeogenesis%=AverageO18FEO18plasma water enrichment×100.

### 2.9 GC–MS of DBSs and data analysis

A 6.5‐mm diameter circle from the DBS was placed in an Eppendorf tube, to which 40 μl of water was added, followed by 400 μl of ethanol. The solution was agitated for 45 min at room temperature then centrifuged at 9300 × *g* for 5 min with a benchtop Eppendorf centrifuge. The supernatant was then transferred to 0.5‐ml Reacti‐therm V‐vials and thoroughly dried. For derivatization of glucose to the aldonitrile pentapropionate, the dried extract was reconstituted in 50 μl of hydroxylamine hydrochloride in pyridine (20 mg/ml) and agitated with a micro stir bar at 90°C for 1.5 h. One hundred microliters of propionic anhydride was then added to each vial and agitated for 30 min at 70°C.^20^ After derivatization, the samples were dried under N_2_ and reconstituted in 100 μl of ethyl acetate. The samples were analyzed with an ISQ Single Quadrupole Mass Spectrometer and Trace 1310 Gas Chromatograph (Thermo Scientific, Waltham, MA, USA) with the following parameters: start temperature of 80°C with a hold time of 1 min followed by 20°C per min ramp up to 280°C, and a 5‐min bakeout at 280°C. The transfer line and ion source temperature were maintained at 280 and 230°C, respectively. The solvent delay on GC–MS was set to 10 min and an m/z filter of 40–600 m/z was used. The retention time of the derivative was 11.5 min. Hard ionization at 70 eV produced the following fragments: m/z 284.11 containing positions 2, 3, and 4 of the original glucose; m/z 370.15 containing positions 2, 3, 4, and 5 of the original glucose; m/z 259.11 containing positions 4, 5, and 6 of the original glucose; and m/z 173.00 containing positions 5 and 6 of the original glucose. Individual isotopologues were identified and quantified by area using their known m/z ions. The isotopic ^18^O ratio for a given fragment ion was calculated as the ratio of the intensity of each mass isotopologue to the sum of all mass isotopologues. The isotopologue distribution pattern was then corrected for natural abundance using Isotopomer Network Compartment Analysis software.^21^ The average ^18^O‐enrichment per position for a given fragment was calculated from the excess M + 2 and M + 4 enrichments and gluconeogenic fractions were calculated as described for the LC–MS/MS data.

Glycerol obtained from triglyceride transesterification was dissolved in 50 μl of pyridine and incubated at 30°C for 30 min in a Reacti‐Therm vial. Fifty microliters of MTBSTFA + 1% TBDMS reagent was added to the vial and incubated at 60°C for 1 h. The reaction solution was transferred into a sample vial and GC–MS analysis was performed using the same instrument as for the glucose aldonitrile pentapropionate derivative. The following parameters were used for GC–MS analysis of glycerol 3‐TBDMS derivative: the initial oven temperature of GC was 60°C for 1 min followed by a ramp of 10°C/min up to 325°C and finally a hold time of 5 min.^22^ The isotopic ^18^O and natural abundance correction was performed as for the glucose aldonitrile pentapropionate fragments.

All comparisons of ^18^O enrichments between different glucose‐6‐phosphate positions and between diets were evaluated by the Mann–Whitney test. The significance level was set as two‐sided with α = 0.05.

## 3 RESULTS

### 3.1 Analysis of glycogen ^18^O‐enrichment by ^13^C NMR

**FIGURE 2:**
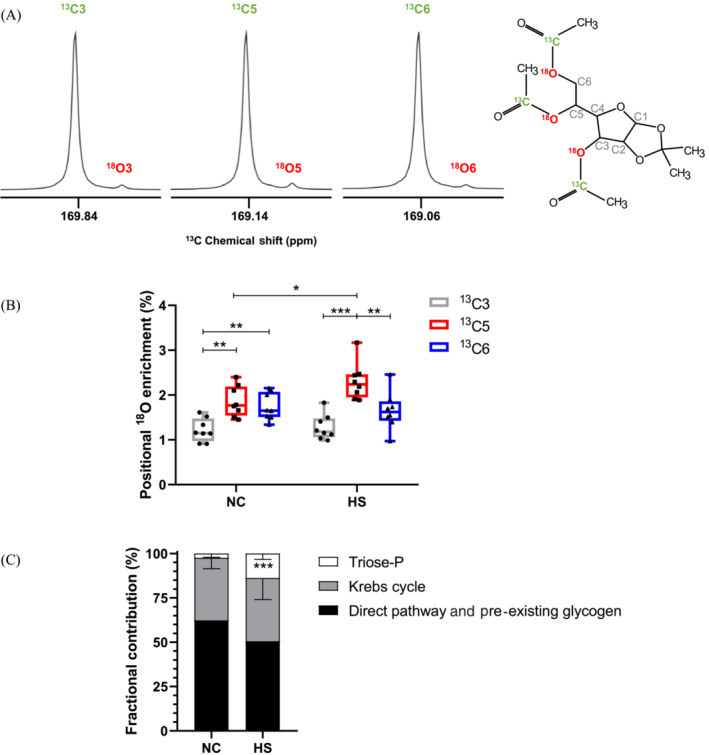
Enrichment of liver glycogen of mice administered with H_2_^18^O as measured by ^13^C NMR spectroscopy of the TAMAG derivative and sources of glycogen estimated from ^18^O‐enrichments of positions 5, 6, and body water. (A) Representative ^13^C signals of the ^13^C‐enriched reporter acetyl carboxyls bound to oxygens 3, 5, and 6 of the hexose moiety with the upfield ^18^O‐shifted signals; (B) Boxplot showing ^18^O excess enrichments of glycogen positions 3, 5, and 6 for mice fed normal chow (NC) and NC supplemented with sugar (i.e., a high sugar [HS] diet); and (C) Estimates of the fractional contributions of triose phosphate sources, Krebs cycle sources, and direct pathway plus pre‐existing glycogen for NC and HS mice (mean ± standard deviation). For both (B) and (C), *p ≤ 0.05, **p ≤ 0.01, and ***p ≤ 0.001 (Mann–Whitney test). TAMAG, 3,5,6‐tri‐O‐[1‐^13^C]acetyl monoacetone glucose

Both groups of mice had similar liver glycogen content (146 ± 27 μmol/g wet weight NC and 117 ± 34 μmol/g wet weight HS) and the ^18^O‐enrichment levels of plasma water were also similar (4.75% ± 0.32% NC and 4.95% ± 0.51% HS). The TAMAG derivatives of liver glycogen presented intense and narrow signals for the ^13^C‐enriched carboxyls bound to oxygens 3, 5, and 6 of the TAMAG hexose moiety with well‐resolved ^18^O‐shifted signals (chemical shift distance between ^16^O and ^18^O signals of 2.65, 2.47, and 2.47 Hz, respectively) (Figure 2A). The positional ^18^O‐enrichments estimated from these signals are shown in Figure 2B. Excess ^18^O‐enrichments ranged from 1.5% to 3.0%, and were not uniformly distributed among positions 3, 5, and 6 for either of the NC or HS groups. Within each group, position 5 enrichment was significantly higher compared with both 6 and 3, while enrichment of position 6 was significantly higher than that of 3. Between each group, position 5 enrichment of the HS group was significantly higher compared with NC and the difference between position 5 and 6 enrichments was significantly higher for HS compared with NC diet (0.12 ± 0.10 NC vs. 0.65 ± 0.16 HS, *p* = 0.0002). The sources of hepatic glycogen synthesis as calculated from the ^18^O‐enrichments of positions 5, 6, and plasma water are shown in Figure 2C. HS mice had significantly higher fractions of glycogen derived via the indirect pathway that was sustained by significant increases in the contributions of substrates entering at the level of triose phosphates.

### 3.2 Blood glucose analysis by GC–MS, LC–MS/MS, and ^13^C NMR

TAMAG derivatives prepared from blood glucose yielded ^18^O‐shifted signals with poorer signal‐to‐noise ratios compared with those prepared from glycogen because of the lower quantities of the analyte. The ^18^O‐enrichment distributions in positions 3, 5, and 6 resemble those seen in glycogen, with position 5 being more highly enriched than either 6 or 3 (Figure S1). The ^18^O‐enrichment values were also more dispersed compared with those of liver glycogen, therefore the differences in positional ^18^O‐enrichments within and between the groups were less well defined. Overall, the enrichment levels of plasma glucose were systematically higher compared with those of glycogen. This may in part be explained by incomplete turnover of liver glycogen during the overnight feeding period, leading to dilution of the ^18^O‐enrichments by pre‐existing unlabeled glycogen.

**FIGURE 3:**
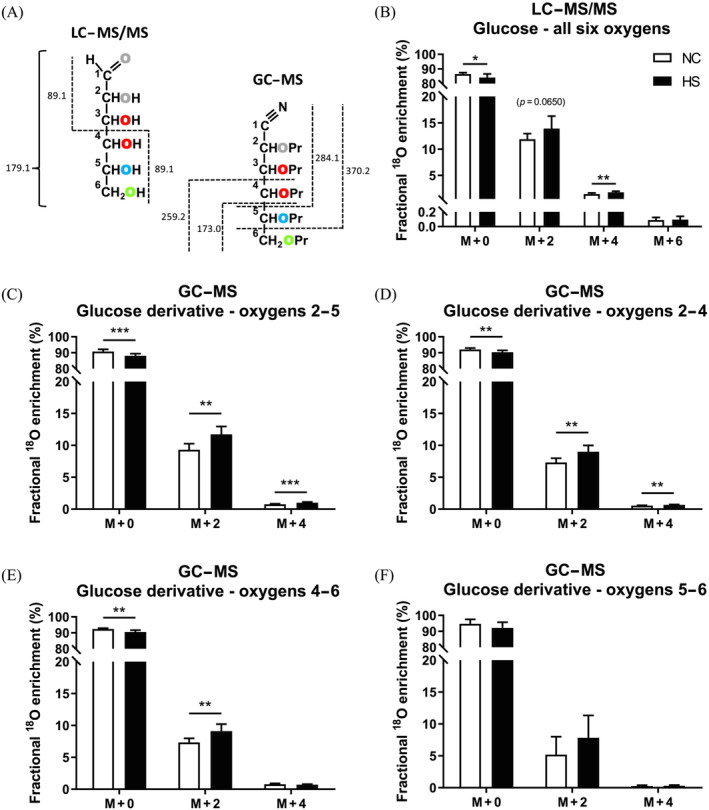
Blood glucose mass isotopomer distributions (mean ± standard deviation) dried blood spots analysis following ^18^O‐administration to mice fed normal chow (NC) and NC supplemented with sugar (i.e., a high sugar [HS] diet). (A) Schematic overview of precursor and fragments obtained from LC–MS/MS analysis of glucose with representation of unlabeled glucose precursor (m/z 179) that originates two fragments with m/z 89 and positional information obtained from GC–MS fragment analysis of glucose aldonitrile pentapropionate derivatives with representation of unlabeled glucose fragments m/z 173, 259, 284, and 370. Pr – propionate group (C_3_H_5_O). (B–F) Analysis of dried blood spots by (B) LC–MS/MS of all six glucose oxygens and (C) GC–MS of derivatized glucose fragments containing Oxygens 2–5, (D) Oxygens 2–4, (E) Oxygens 4–6, and (F) Oxygens 5–6. *p ≤ 0.05, **p ≤ 0.01, and ***p ≤ 0.001 (Mann–Whitney test). GC–MS, gas chromatography mass spectrometry; LC–MS/MS, liquid chromatography coupled to tandem mass spectrometry

MS data acquired from DBSs of NC and HS mice are summarized in Figure 3. For the LC–MS/MS data gathered from the entire glucose molecule, the expected M + 2 contribution was accompanied by progressively lower amounts of M + 4 and M + 6 signals representing glucose molecules containing two and three ^18^O, respectively. These observations reflect the fact that ^18^O‐enrichment of a particular precursor site occurs independently of all other sites, hence leading to the possibility of multiple ^18^O‐enriched glucose molecules. The GC–MS data from various glucose fragments (Figure 3B–E) also featured detectable M + 4 signals, representing the presence of two ^18^O, alongside the expected M + 2 signals. The fragmentation procedure generated lesser amounts of the 5–6 fragment compared with the other three (data not shown) and this probably contributed to the higher uncertainty in ^18^O‐enrichment quantification for this fragment compared with the others. Table 1 shows the calculated average fractional ^18^O‐enrichments from the GC–MS and LC–MS/MS data. For NC mice, the LC–MS/MS and GC–MS fragmentation data gave highly consistent estimates, but the 5–6 fragment data had much higher variance compared with the others. For both NC and HS mice, the average enrichment of the six glucose oxygens estimated by LC–MS/MS was significantly lower compared with that of oxygens 2–4 and 4–6 estimated by GC–MS and was also significantly lower than that of oxygens 2–5 for HS mice. The average ^18^O‐enrichment estimates from the 5–6 fragment did not show a statistical difference between NC and HS, nor did they differ from the LC–MS/MS determinations for either condition, presumably because of the high variance in 5–6 ^18^O‐enrichment data.

**TABLE 1:** Mean ^18^O‐enrichment of selected plasma glucose oxygens as measured by LC–MS/MS transitions representing oxygens 1–6, and GC–MS fragments representing oxygens 2–5, 2–4, 4–6, and 5–6 for a group of eight mice fed NC and a second group of eight mice fed a HS diet. Data are normalized to the number of oxygens in each glucose species. Also shown is the plasma water ^18^O‐enrichment for both groups and estimates of the gluconeogenic contribution to blood glucose appearance calculated from the ratio of mean ^18^O‐enrichment for each group of glucose oxygens to that of body water (bold text). The values shown are means ± standard deviation

Experimental group	Mean plasma glucose ^18^O‐enrichment for selected oxygens (%)					Plasma water ^18^O‐enrichment (%)
	Fraction of blood glucose derived from gluconeogenesis (%)					
	*LC–MS/MS*	*GC–MS*	*GC–MS*	*GC–MS*	*GC–MS*	
	*All 6 oxygens*	*Oxygens 2, 3, 4, 5*	*Oxygens 2, 3, 4*	*Oxygens 4, 5, 6*	*Oxygens 5, 6*	
**NC**	2.5 ± 0.2	2.7 ± 0.3	2.8 ± 0.2^**†**^	3.0 ± 0.2^††^, ^†^	2.9 ± 1.5	4.9 ± 0.3
	**51 ± 5**	**55 ± 8**	**57 ± 8**	**60 ± 9** ^**†**^	**58 ± 29**	
**HS**	3.0 ± 0.5^$^	3.4 ± 0.4^#^, ^**†**^	3.4 ± 0.4^#^, ^**†**^	3.5 ± 0.4^#^, ^**†**^	4.2 ± 1.9	4.7 ± 0.5
	**63 ± 14** ^$^	**73 ± 12** ^$^	**73 ± 13** ^$^	**75 ± 13** ^$^, ^**†**^	**93 ± 54**	

Abbreviations: GC–MS, gas chromatography mass spectrometry; HS, high sugar; LC–MS/MS, liquid chromatography coupled to tandem mass spectrometry; NC, normal chow.

$ *p* ≤ 0.05 compared with NC (Mann–Whitney test).

# *p* ≤ 0.01 compared with NC (Mann–Whitney test).

^**†**^ *p* ≤ 0.05 compared with LC–MS/MS (Mann–Whitney test).

^**††**^ *p* ≤ 0.01 compared with LC–MS/MS (Mann–Whitney test).

Estimates of the gluconeogenic fraction of plasma glucose based on the ratio of average ^18^O‐enrichment to that of body water showed significantly higher values for HS compared with NC diet, with the exception of the values obtained by GC–MS analysis of the 5–6 fragment (Table 1, bold numbers). Within each diet, the gluconeogenic fraction estimated by LC–MS/MS was significantly lower than that estimated by GC–MS for oxygens 4–6, and for the HS diet, it tended to be lower compared with the estimates from GC–MS analysis of oxygens 2–4 and 2–5 (*p* = 0.065 for each fragment).

### 3.3 Liver triglyceride glycerol ^18^O‐enrichment analysis by GC–MS

The average ^18^O‐enrichment of liver triglyceride glycerol oxygens as measured by GC–MS of three different fragments is shown in Table 2. Overall, the average glycerol ^18^O‐enrichment levels tended to be systematically higher compared with those measured in plasma glucose and indicate a high turnover rate of hepatic triglyceride glycerol during the overnight interval. Unlike plasma glucose, the average glycerol ^18^O‐enrichment levels did not significantly differ between NC and HS mice for any of the fragments studied. However, the distribution of ^18^O‐enrichment among the three glycerol oxygens was less even for HS compared with NC mice, as seen by a significantly higher enrichment of the 1–2 (2–3) compared with the 1 (3) fragments.

**TABLE 2:** Mean ^18^O‐enrichment of liver triglyceride glycerol oxygens as measured by GC–MS fragments representing oxygen 1 or 3, oxygen 1–2 or 2–3, and oxygen 1–3 for a group of eight mice fed NC and a second group of eight mice fed a HS diet. Also shown are estimates of fractional triglyceride glycerol turnover obtained from the ratio of the mean ^18^O‐enrichment of oxygens 1, 2, and 3 relative to that of body water. The values shown are means ± standard deviation

Experimental group	Mean liver triglyceride glycerol ^18^O‐enrichment (%)			Fractional glycerol turnover (%)
	*GC–MS*	*GC–MS*	*GC–MS*	
	*Oxygen 1 (3)*	*Oxygens 1, 2 (2, 3)*	*Oxygens 1, 2, 3*	
**NC**	3.4 ± 0.4	3.2 ± 1.0	3.6 ± 0.2	77 ± 8
**HS**	3.1 ± 0.5	3.8 ± 0.5^$^	3.4 ± 0.4	70 ± 7

Abbreviations: GC–MS, gas chromatography mass spectrometry; HS, high sugar; NC, normal chow.

$ *p* = 0.016 compared with HS oxygen 1 (3) (Mann–Whitney test).

## 4 DISCUSSION

^18^O‐enriched water was administered to mice and the incorporation of ^18^O into synthesized glucose and glycogen was analyzed by ^13^C NMR, LC–MS/MS, and GC–MS. H_2_^18^O presents some advantages over ^2^H_2_O for metabolic studies, the most important of which is an absence of significant isotope effects associated with ^18^O metabolism compared with ^2^H. KIE associated with ^2^H not only confound modeling of metabolite enrichment data but also induce significant toxic effects, and even death, when body water ^2^H‐enrichment exceeds 30%.^23^ With ^18^O, no adverse effects were found for mice that breathed 90% ^18^O_2_ for three generations with the third generation also drinking H_2_^18^O enriched to 90%.^24^

Following administration, H_2_^18^O is diluted by water (including water generated by respiration) and additionally by respiratory CO_2_ and exchange of its oxygens with those of water, while ^2^H_2_O is diluted by water alone. This provides the basis for estimating whole‐body respiration by measuring the differential dilution of ingested ^2^H_2_O and H_2_^18^O.^25^ In mice, daily CO_2_ production represents about 20% of the total oxygen content of body water.^26^ Therefore, compared with the dilution of ^2^H following administration of a ^2^H_2_O bolus, the same quantity of H_2_^18^O would be expected to be additionally diluted by ~20%. This may explain the observed body water enrichment of ~5%, despite administering a bolus of ~8/100 ml of body water H_2_^18^O coupled with a maintenance enrichment of 10% in the drinking water.

Under these conditions, excess ^18^O‐enrichment levels of plasma glucose and liver glycogen were in the range of 1.5%–4.0%. For the isotope‐shifted ^13^C NMR measurement of ^18^O‐enrichment, these values are at the limit of precise quantification, even with the ^13^C‐enriched TAMAG derivative. TAMAGs prepared from glycogen had superior signal‐to‐noise ratios over those obtained from glucose and for this reason demonstrated more consistent and statistically significant differences in excess ^18^O‐enrichment between positions 3, 5, and 6. Because hepatic glucose‐6‐phosphate is the precursor for glycogen as well as the bulk of endogenously generated glucose, it is expected that plasma glucose and glycogen should have concordant ^18^O‐enrichment patterns (although absolute ^18^O‐enrichment levels of the two metabolites could have systematic differences because of the slower turnover rate of liver glycogen compared with plasma glucose). The ^18^O‐enrichment distributions in positions 5 and 6 of liver glycogen relative to that of body water yielded information on indirect pathway contributions and sources that are in reasonable agreement with previously published ^2^H enrichments of the same positions for mice fed NC and HS diets over a 6‐week period.^27^ The elevated contribution of triose‐P precursors in HS mice reflects the glycogenic metabolism of the fructose component present in their diet.

For analysis of blood glucose ^18^O‐enrichment, ^13^C NMR analysis of the largest possible quantity of blood that could be harvested posteuthanization from each mouse (~1.0 ml) was limited by the quality of the spectra and signal‐to‐noise ratios of the ^18^O‐shifted signals. For LC–MS/MS and GC–MS analyses of glucose recovered from DBSs representing a few microliters of whole blood, signal limitation was not a factor, with the exception of the GC–MS fragment containing glucose oxygens 5 and 6. GC–MS analysis of the pentapropionyl aldonitrile glucose derivative yielded data from several fragments representing oxygens 2–6 of glucose. Although an early study demonstrated the analysis of ^18^O enrichments from various ^18^O‐glucose standards via pentaacetyl glucose,^28^ we chose pentapropionyl aldonitrile based on our past experience with preparation and analysis of this derivative and the fact that it generated the expected fragmentation data with our ^18^O‐glucose standards (data not shown). Oxygen 2 of glucose‐6‐phosphate can become enriched by the anomerization of fructose‐6‐phosphate, an exchange process that is independent of gluconeogenesis, while oxygen 1 enrichment can be modified by glucose anomerization in vivo as well as in vitro.^29^ On this basis, the exclusion of glucose oxygen 1 and 2 enrichment information would be expected to provide a better assessment of gluconeogenic activity. Nevertheless, for NC mice, the average ^18^O‐enrichment of all six oxygens measured by LC–MS/MS was highly consistent with the average enrichments of each of the four glucose fragments measured by GC–MS. However, for HS mice, the average ^18^O‐enrichment determined by LC–MS/MS was consistently lower compared with those measured with GC–MS. This may in part be explained by fructose gluconeogenesis in HS, resulting in ^18^O‐enrichment of oxygens 2–5 but not oxygens 1 and 6. Moreover, glucose oxygen 1 enrichment could have undergone depletion poststudy by spontaneous exchange with water via anomerization.

Blood glucose analysis revealed that gluconeogenesis also accounted for a substantial fraction of postprandial circulating glucose for both the NC and HS groups. This has also been previously reported in rats that were studied under similar postprandial conditions with high sucrose and NC diets,^30^ as well as in fasted rats that were given an intraperitoneal glucose load,^31^ and following protein meals supplemented with different mixtures of glucose and fructose in overnight‐fasted healthy humans.^32^ There was a significantly increased gluconeogenic contribution to postprandial glucose levels for HS compared with NC mice, consistent with the gluconeogenic conversion of fructose to glucose. In the study conducted by Barosa et al., the postprandial gluconeogenic fraction was significantly higher in healthy subjects following a protein meal supplemented with high fructose (55% fructose/45% glucose) compared with a meal supplemented with 5% fructose and 95% glucose.^32^ However, in rats whose NC was supplemented with sucrose in the drinking water, the total gluconeogenic contribution was not significantly different from rats fed NC alone, but the sources of gluconeogenic carbons were significantly shifted from anaplerotic precursors to those entering at the triose phosphate level, which include fructose.^30^

The triglyceride glycerol is derived from dihydroxyacetone phosphate via glycerol‐3‐phosphate (Figure 1), but following recovery via transesterification and derivatization for GC–MS analysis, the identity of positions 1 and 3 are no longer retained. Hence, enrichments of positions 1 (3) reflect the average enrichment of dihydroxyacetone phosphate (DHAP) positions 1 and 3, while position 2 enrichment translates to DHAP position 2. Because ^18^O‐enrichment of the three DHAP oxygens is independent of its formation from glycolysis or gluconeogenesis, the ^18^O‐enrichment distribution of glycerol does not inform the contributions of glycolysis and glyceroneogenesis to glycerol‐3‐phosphate formation. Instead, we found that the ^18^O‐enrichment distribution was weighted more towards position 2 compared with positions 1 and 3 when NC was supplemented by sugar. This observation is consistent with a high contribution of fructose carbons to glycerol‐3‐phosphate synthesis, where position 2 of the DHAP precursor is quantitatively enriched from ^18^O‐water via aldolase, while DHAP position 3 is not enriched. In mice fed NC supplemented with a 50/50 mix of ^13^C‐enriched fructose and glucose, the fructose component accounted for over half of the newly synthesized triglyceride glycerol, while fractional triglyceride glycerol turnover as measured with ^2^H_2_O showed a wide variability, ranging from 20% to 80% with a mean of ~40%.^17^ By comparison, the ^18^O‐enrichment measurements yielded turnover rates of ~70%, with no detectable differences between NC and HS. This difference may reflect a higher degree of ^18^O‐equilibration between body water and triose‐P precursors compared with that of ^2^H, in particular during the conversion of fructose to glycerol‐3‐phosphate. If so, then H_2_^18^O may provide a more precise estimate of triglyceride glycerol turnover compared with ^2^H_2_O. H_2_^18^O has also been used to measure the turnover of the acyl moieties of membrane phospholipids of peritoneal exudate cells in culture based on carboxyl and water oxygen exchange.^33^ However, for the triglycerides in the current study, ^18^O‐enrichments of the fatty acyl oxygens were assumed to have undergone extensive exchange with those of the reagents during transesterification, hence analysis of the fatty acyl ^18^O‐enrichments was not performed.

There are some important limitations to the use of H_2_^18^O for in vivo measurements of carbohydrate metabolism compared with ^2^H_2_O. Not only is the current cost of H_2_^18^O much higher than that of ^2^H_2_O, but the reliable quantification of ^18^O‐shifted ^13^C signals requires higher field NMR instruments (800 MHz) compared with those required for quantifying ^2^H signals of derivatized glucose (≥ 500 MHz). While there is no toxicity associated with any level of body ^18^O‐water enrichment, there are limitations to the extent to which ^18^O‐body water enrichment can be acutely increased by gavage, infusion, or i.p. injection of H_2_^18^O. This is particularly relevant when applying H_2_^18^O to studies of endogenous glucose sources during fasting, where the interval between labeled water administration and sampling of blood glucose may be only a few hours.

In conclusion, ^18^O‐enrichment of gluconeogenic precursors at the level of triose phosphates was inferred from NMR and MS analyses of liver glycogen and plasma glucose. Positional enrichment distributions, as measured from ^18^O isotope‐shifted ^13^C NMR signals, were in accordance with the known enzymatic and nonenzymatic mechanisms for ^18^O‐exchange between water and triose‐phosphate oxygens and yielded estimates of indirect pathway contributions from the Krebs cycle and triose phosphate sources to glycogenesis. For the analysis of blood glucose ^18^O‐enrichments, the NMR measurement was limited by low signal‐to‐noise ratios for the small ^18^O isotope‐shifted carbon signals. Both GC–MS and LC–MS/MS provided robust quantification of blood glucose ^18^O‐enrichment from a few microliters of dried blood, with GC–MS providing partial resolution of ^18^O‐positional enrichments through analysis of glucose fragments (Figure 3). For LC–MS/MS, the transitions chosen for observation did not provide positional ^18^O information. However, if [3‐^18^O]‐ and [4‐^18^O]glucose standards become commercially available for formulating isotope calibration curves, then other observed LC–MS/MS transitions that read the glucose 3–6 oxygens can be exploited to provide more precise estimates of gluconeogenesis and substrate contributions.

### CONFLICTS OF INTEREST

The authors declare no competing interests.

## AUTHOR CONTRIBUTION

JGJ, MC, GDB, and MEM designed the experiments. MC, JGJ GDB, IV, RM, AG, AT, VMM, and MEM conducted the experiments. MEM, BM, JGJ, and AG provided facilities to perform the experiments and provided materials and instrumentation to perform the experiments and analyze samples. Results were discussed and analyzed by MC, JGJ, RM, and MEM. The manuscript was written by MC, JGJ, RM, and MEM. All authors reviewed the manuscript.

## Supporting information


**Table S1:** Multiple reaction monitoring (MRM) transitions (m/z for Q1 and Q3) of the LC–MS/MS data acquisition method for glucose and ^18^O labelled glucoses.
**Figure S1:** Box‐plot showing ^18^O excess enrichments of plasma glucose positions 3, 5 and 6 as measured by ^13^C NMR spectroscopy of the TAMAG derivative for mice fed normal chow (NC) and chow supplemented with sugar (HS). * *p* ≤ 0.05 (Mann–Whitney test).
