# Supplementary material for: Enrichment of hepatic glycogen and plasma glucose from H₂18O informs gluconeogenic and indirect pathway fluxes in naturally feeding mice
Source: NMR Biomed. 2022 Oct 22;36(2):e4837. doi: 10.1002/nbm.4837 (PMC9845176; doi:10.1002/nbm.4837)
Supplement: Supplementary file 1 — Table S1: Multiple reaction monitoring (MRM) transitions (m/z for Q1 and Q3) of the LC–MS/MS data acquisition method for glucose and 18O labelled glucoses. Figure S1: Box‐plot showing 18O excess enrichments of plasma glucose positions 3, 5 and 6 as measured by 13C NMR spectroscopy of the TAMAG derivative for mice fed normal chow (NC) and chow supplemented with sugar (HS). * p ≤ 0.05 (Mann–Whitney test). [file NBM-36-0-s001.docx]

**Supplementary material of** **“Enrichment of Hepatic Glycogen and Plasma Glucose from H_2_^18^O Informs Gluconeogenic and Indirect Pathway Fluxes in Naturally-feeding Mice”**

| **Supplementary table 1 -** Multiple reaction monitoring (MRM) transitions (m/z for Q1 and Q3) of the LC-MS/MS data acquisition method for glucose and ^18^O labelled glucoses. |
| --- |
| \|  \| **Metabolite** \| **Q1** \| **Q3** \| **CXP** \| \| --- \| --- \| --- \| --- \| --- \| \| 1 \| Glucose \| 179.1 \| 89.1 \| -3.0 \| \| 2 \| Glucose \| 179.1 \| 119.1 \| -7.0 \| \| 3 \| Glucose M+2 (1 ^18^O) \| 181.1 \| 89.1 \| -5.0 \| \| 4 \| Glucose M+2 (1 ^18^O) \| 181.1 \| 91.1 \| -1.0 \| \| 5 \| Glucose M+4 (2 ^18^O) \| 183.1 \| 89.1 \| -1.0 \| \| 6 \| Glucose M+4 (2 ^18^O) \| 183.1 \| 91.1 \| -3.0 \| \| 7 \| Glucose M+4 (2 ^18^O) \| 183.1 \| 93.1 \| -7.0 \| \| 8 \| Glucose M+6 (3 ^18^O) \| 185.1 \| 89.1 \| -1.0 \| \| 9 \| Glucose M+6 (3 ^18^O) \| 185.1 \| 91.1 \| -1.0 \| \| 10 \| Glucose M+6 (3 ^18^O) \| 185.1 \| 93.1 \| -3.0 \| \| 11 \| Glucose M+6 (3 ^18^O) \| 185.1 \| 95.1 \| -7.0 \| \| 12 \| Glucose M+8 (4 ^18^O) \| 187.1 \| 91.1 \| -1.0 \| \| 13 \| Glucose M+8 (4 ^18^O) \| 187.1 \| 93.1 \| -3.0 \| \| 14 \| Glucose M+8 (4 ^18^O) \| 187.1 \| 95.1 \| -7.0 \| \| 15 \| Glucose M+10 (5 ^18^O) \| 189.1 \| 93.1 \| -3.0 \| \| 16 \| Glucose M+10 (5 ^18^O) \| 189.1 \| 95.1 \| -7.0 \| \| 17 \| Glucose M+12 (6 ^18^O) \| 191.1 \| 95.1 \| -7.0 \| \| 18 \| [U-^13^C_6_, ^2^H_7_]glucose (IS) \| 192.1 \| 94.1 \| -7.0 \| \| 19 \| [U-^13^C_6_, ^2^H_7_]glucose (IS) \| 192.1 \| 128.1 \| -7.0 \|   Note: Collision cell exit potential (CXP), internal standard (IS) |


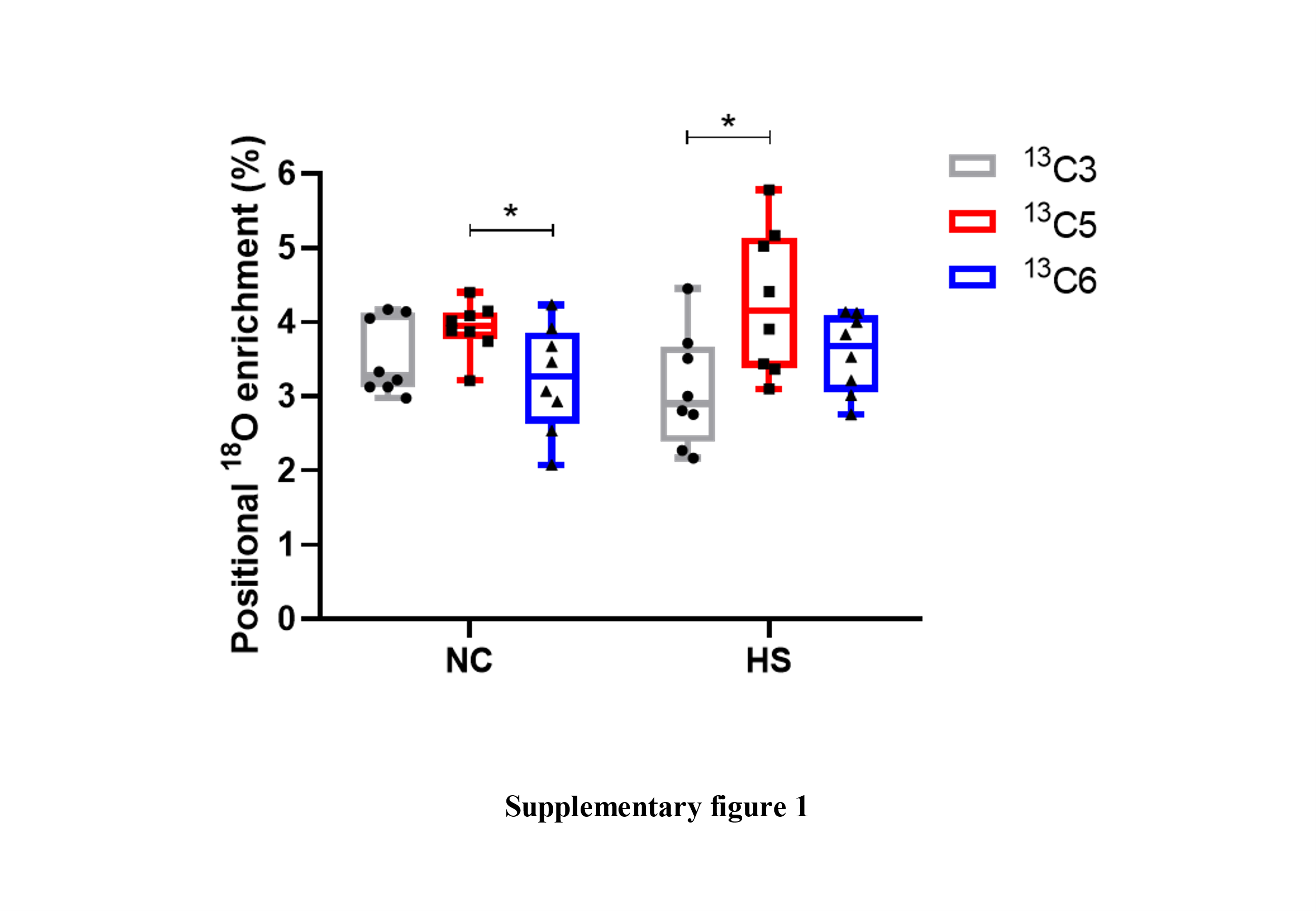


**Supplementary figure 1:** Box-plot showing ^18^O excess enrichments of plasma glucose positions 3, 5 and 6 as measured by ^13^C NMR spectroscopy of the TAMAG derivative for mice fed normal chow (NC) and chow supplemented with sugar (HS). * *p* ≤ 0.05 (Mann-Whitney test).
